# Data in flood risk assessment of metro systems in a subsiding environment using the interval FAHP–FCA approach

**DOI:** 10.1016/j.dib.2019.104468

**Published:** 2019-09-03

**Authors:** Hai-Min Lyu, Shui-Long Shen, Annan Zhou, Wan-Huan Zhou

**Affiliations:** a Key Laboratory of Intelligent Manufacturing Technology (Shantou University), Ministry of Education, Shantou, Guangdong 515063, China; b Department of Civil and Environmental Engineering, College of Engineering, Shantou University, Shantou, Guangdong 515063, China; c Civil and Infrastructure Engineering Discipline, School of Engineering, Royal Melbourne Institute of Technology (RMIT), Victoria 3001, Australia; d Department of Civil and Environmental Engineering, University of Macau, Macau S.A.R., China

**Keywords:** FAHP, FCA, Flood risk, Subsiding environment, Metro system

## Abstract

Floods in the metro system have caused catastrophic damages in mega-cities, especially in subsiding environment. This data in brief gives a detailed description for the calculation of judgment matrixes during decision making process for the flood risk assessment of the metro system. The data source of the assessment factors is provided. The analytical hierarchy process (AHP) and interval fuzzy AHP (FAHP) are used to calibrate the weights of assessment factors. The fuzzy clustering analysis (FCA) method is used to modify the weights obtained from AHP and interval FAHP. The data presented herein was used for the article, titled “Flood risk assessment of metro systems in a subsiding environment using the interval FAHP–FCA approach” Lyu et al. (2019) [1].

**Table:** Specifications Table

Subject area	Civil engineering
More specific subject area	Environmental geotechnical engineering
Type of data	Table
How data was acquired	The data was produced by reanalyzing data from the following websites: http://www.shanghai.gov.cn/; http://data.cma.cn/; http://www.gscloud.cn/; https://map.baidu.com/.
Data format	Raw, analyzed
Experimental factors	The data were processed with 30 m resolution in GIS before analysis.
Experimental features	The data were collected from the website of local government and the statistic yearbook of Shanghai (see Table 2).
Data source location	Shanghai, China
Data accessibility	Data are included in this article
Related research article	Lyu, H.M., Shen, S.L., Zhou, A.N., Zhou, W.H. Flood risk assessment of metro systems in a subsiding environment using the interval FAHP–FCA approach, Sustainable Cities and Society, 2019, 50, 101682, https://doi.org/10.1016/j.scs.2019.101682.

**Table:** 

**Value of the data** • The data sources of the all assessment factors was provided. • The judgment matrixes of AHP and interval FAHP express the opinions from decision makers during pairwise comparison process. • The calculated weights of each assessment index are used to take overlay analysis in GIS. • The calculation process can help researchers to understand how to apply FAHP and FCA methods.

## 1 Data

**Fig. 1:**
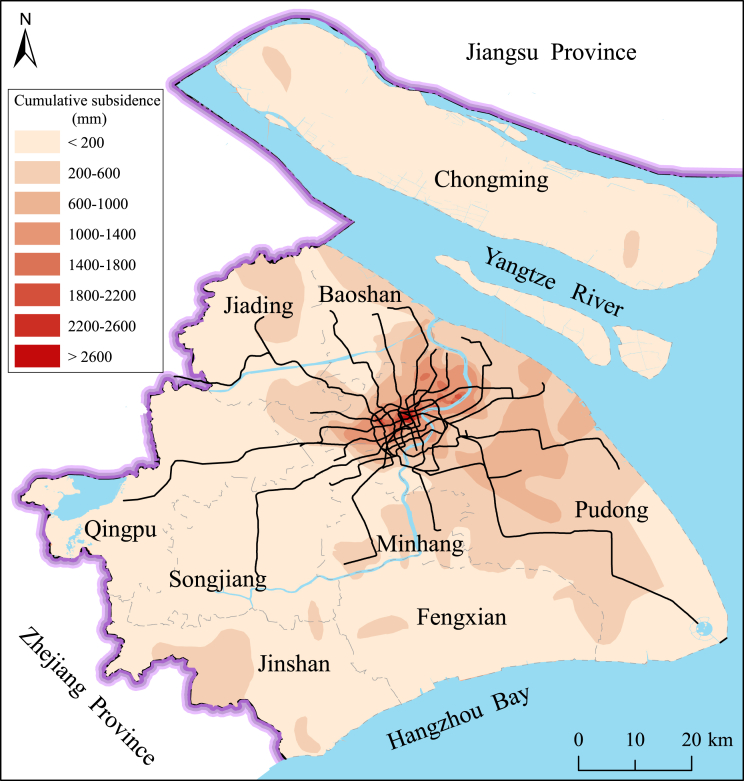
Metro line distribution in subsiding environment.

The data including hazard, exposure and vulnerability. The data presented here was used to calculate the weight coefficients of AHP and interval FAHP methods for the flood risk of metro system in subsiding environment. Fig. 1 shows the distribution of metro system in subsiding environment. The detailed information of hazard data and exposure data can be found in the research article by Lyu et al. (2019) [1]. The vulnerability data is presented in Table 1. The vulnerability data were collected from Shanghai Statistics Bureau [2] and Wang et al. (2014) [3]. During the application of the AHP and interval FAHP, the FCA method is used to classify the assessment sample and modify the weights [5], [6]. According to Saaty (1977) [7], the value of average random consistency index (*RI*) of AHP method is listed in Table 2. Table 3 tabulates the data sources of each assessment factor. Table 4 tabulates the assessment criteria of vulnerability index in district division for Shanghai administrative region.

**Table 1:** Data of vulnerability index in district division for Shanghai administrative region (Data from SSB, 2017 [2] and Wang et al., 2014 [3]).

District	V_1_ (×10^3^p/km^2^)	V_2_ (billion/km^2^)	V_3_ (%)	V_4_ (%)	V_5_ (km/km^2^)	V_6_ (km/km^2^)	V_7_ (km)	V_8_ (×10^3^ rmb/km^2^)
Urban center	24.07	7.48	18.83	22.63	1.03	1.59	86.60	383.8
Pudong	4.55	7.67	24.12	22.93	0.43	0.44	174.96	192.5
Minhang	6.85	8.50	38.56	20.08	0.32	0.2	87.10	72.2
Jiading	3.40	11.54	19.13	18.57	0.12	0.03	0	84.2
Baoshan	7.49	6.61	37.26	25.13	0.28	0.4	169.16	230.9
Songjiang	2.91	5.52	16.72	19.45	0.09	0	0	20.1
Jinshan	1.37	2.67	8.20	15.81	0	0	26.06	47.2
Qingpu	1.81	2.31	9.51	17.44	0	0	0	30.7
Fengxian	1.70	2.08	8.89	15.99	0	0	112.86	31.6
Chongming	0.59	0.30	2.06	25.96	0	0	215.83	37.5

**Table 2:** Value of average random consistency index (*RI*).

n	1	2	3	4	5	6	7	8	9
*RI*	0	0	0.52	0.89	1.12	1.26	1.36	1.41	1.46

**Table 3:** Data sources and description of each factor.

Index	Sub-index	Description	Data source and format
*H*_*i*_	*H*_1_	Maximum daily rainfall	Data from National Meteorological Information Center; visualized with 10 m resolution in GIS
	*H*_2_	Rainfall days with daily rainfall (DR) in excess of 150 mm (DR > 150 mm)	
	*H*_3_	Rainfall days with daily rainfall (DR) in excess of 150 mm (DR > 150 mm)	
	*H*_4_	Annual average rainfall	
	*H*_5_	Regional land subsidence	Author's research result with 30 m resolution
*E*_*j*_	*E*_1_	Number of exits	Data extracted from Baidu Map
	*E*_2_	Type of exit	
	*E*_3_	Step height of the exit	
	*E*_4_	Drainage capacity of the underground space	Metro design standard (GB50157-2013) [4]
	*E*_5_	Elevation of the metro station	Extracted from DEM
	*E*_6_	Longitudinal settlement along the metro lines	Extracted from factor *H*_5_
*V*_*k*_	*V*_1_	Population density	Data from reference SSB (2017) [2]
	*V*_2_	Gross domestic product (GDP) per unit area	
	*V*_3_	Construction land ratio	
	*V*_4_	Green area ratio	
	*V*_5_	Metro line density	
	*V*_6_	Elevated road density	
	*V*_7_	Flood prevention walls	
	*V*_8_	Reduction of flood prevention

**Table 4:** Assessment criterion of vulnerability index in district division for Shanghai administrative region.

Vulnerability index	1	2	3	4	5
V_1_ (×10^3^p/km^2^)	0∼0.8	0.8∼1.0	1.0∼3.0	3.0∼10	10∼25
V_2_ (billion/km^2^)	0∼3	3∼6	6∼8	8∼10	10∼12
V_3_ (%)	0∼8	8∼16	16∼24	24∼32	32∼40
V_4_ (%)	15∼17	17∼19	19∼21	21∼23	23∼26
V_5_ (km/km^2^)	0∼0.05	0.05∼0.1	0.1∼0.2	0.2∼0.4	0.4∼1.1
V_6_ (km/km^2^)	0∼0.02	0.02∼0.1	0.1∼0.2	0.2∼0.6	0.6∼1.6
V_7_ (km)	>200	150∼200	100∼150	30∼100	0∼30
V_8_ (×10^3^rmb/km^2^)	>200	100∼200	80∼100	40∼80	0∼40

Note: p/km^2^ means people in 1 km^2^; b/km^2^ means one billion in 1 km^2^.

## 2 Experimental design, materials and methods

### 2.1 Assessment structure

In the assessment structure, flood risk is the objective layer. The index layer including hazard (*H*_i_), exposure (*E*_j_) and vulnerability (*V*_k_). Each index consists of different sub-indexes (see Table 3). Based on the AHP theory, the judgment matrix can be obtained by pairwise comparison [8], [9]. The interval FAHP uses an interval number instead a crisp number to express decision maker's opinion during pairwise comparison analysis [10].

### 2.2 Weight calibration

In the AHP method, the weight coefficient is calculated by using Eq. (1). In the interval FAHP method, the weight vector of the interval pairwise comparison matrix is calculated from Eq. (2). The detailed description about the equation can be found in the research article (Lyu et al., 2019) [1].

(1) $$w_{i}=\frac{M_{i}}{\sum_{i=1}^{n}M_{i}}$$

where $M_{i}=\sqrt[{n}]{\prod_{j=1}^{n}a_{ij}}$ and *a*_ij_ is the element in the judgment matrix.

(2) $$\vec{w}=\left[w_{1},w_{2}\right]=\left[\alpha w^{-},\beta w^{+}\right]$$

where $\alpha ={\left[\sum_{j=1}^{n}\frac{1}{\sum_{i=1}^{n}a_{ij}^{+}}\right]}^{1/2}$; $\beta ={\left[\sum_{j=1}^{n}\frac{1}{\sum_{i=1}^{n}a_{ij}^{-}}\right]}^{1/2}$; *w*^−^ and *w*^+^ are the weights of the lower bound and upper bound matrixes, respectively, which can be calculated using Eq. (1); *w*_1_ and *w*_2_ are the weights of the interval pairwise comparison matrix.

To elucidate the calculation process of weight coefficients in the FAHP method, the weight calculation process for the index layer to object layer is given as an example. The original AHP approach uses a pairwise comparison to express the relative importance of the assessment factor, as listed in Eq. (3). When the judgment matrix (***R***) meets the consistency requirement, the interval judgment matrix [***R***^−^, ***R***^+^] can then be determined [see Eq. (3)]. The interval fuzzy weights of the hazard, exposure, and vulnerability indices can be obtained, as listed in Eq. (6).

(3) $$\begin{array}{l} R=\left[\begin{array}{ccc} \frac{H}{H} & \frac{H}{V} & \frac{H}{E}\\ \frac{V}{H} & \frac{V}{V} & \frac{V}{E}\\ \frac{E}{H} & \frac{E}{V} & \frac{E}{E} \end{array}\right]=\left[\begin{array}{ccc} 1 & 3 & 2\\ \frac{1}{3} & 1 & \frac{1}{2}\\ \frac{1}{2} & 2 & 1 \end{array}\right]\Rightarrow \left[R^{-},R^{+}\right]=\left[\begin{array}{ccc} 1 & 2.5-3.5 & 1.5-2.5\\ \frac{1}{3.5}-\frac{1}{2.5} & 1 & \frac{1}{3}-\frac{2}{3}\\ \frac{1}{2.5}-\frac{1}{1.5} & \frac{3}{2}-3 & 1 \end{array}\right]\\ \Rightarrow R^{-}=\left[\begin{array}{ccc} 1 & 2.5 & 1.5\\ \frac{1}{3.5} & 1 & \frac{1}{3}\\ \frac{1}{2.5} & \frac{3}{2} & 1 \end{array}\right],R^{+}=\left[\begin{array}{ccc} 1 & 3.5 & 2.5\\ \frac{1}{2.5} & 1 & \frac{2}{3}\\ \frac{1}{1.5} & 3 & 1 \end{array}\right] \end{array}$$

(4) $$\alpha ={\left[\sum_{j=1}^{n}\frac{1}{\sum_{i=1}^{n}a_{ij}^{+}}\right]}^{1/2}=\sqrt{\frac{1}{\left(1+\frac{1}{3.5}+\frac{1}{2.5}\right)}+\frac{1}{\left(2.5+1+\frac{3}{2}\right)}+\frac{1}{\left(1.5+\frac{1}{3}+1\right)}}=0.9259$$

(5) $$\beta ={\left[\sum_{j=1}^{n}\frac{1}{\sum_{i=1}^{n}a_{ij}^{-}}\right]}^{1/2}=\sqrt{\frac{1}{\left(1+\frac{1}{3.5}+\frac{1}{2.5}\right)}+\frac{1}{\left(2.5+1+\frac{3}{2}\right)}+\frac{1}{\left(1.5+\frac{1}{3}+1\right)}}=1.0706$$

(6) $$w_{R}=\left[\alpha w_{R}^{-},\beta w_{R}^{+}\right]=\left[\begin{array}{c} H\\ V\\ E \end{array}\right]=\left[\begin{array}{c} 0.5041,0.5565\\ 0.1301,0.1623\\ 0.2956,0.3403 \end{array}\right]$$

Following the same method, the weight coefficients of other factors can also be obtained.

### 2.3 FCA approach

The FCA approach is used to calibrate the fuzzy clustering matrix (***H***) and fuzzy class centre matrix (***K***) of the assessment sample. In this study, the FCA method is used to identify the vulnerable risk of metro system induced by floods. Table 1 lists the data of vulnerability index of district division for Shanghai administrative region. Table 4 tabulates the assessment criterion of vulnerability index of district division for Shanghai administrative region. Based on these data, the fuzzy clustering matrix (***H***) and fuzzy class centre matrix (***K***) can be obtained by using the FCA method. The result can be found in the research article (Lyu et al., 2019) [1]. The detailed description of the FCA method is as follows.

The generalised Euclidean distance (*d*) is used to calibrate the relative connection degree between the sample *i* and the class *k*, as given in Eq. (7).

(7) $$d=\left\|b_{i}-k_{c}\right\|=\sqrt{\sum_{j=1}^{m}{\left(b_{ij}-k_{jc}\right)}^{2}}$$

where $b_{i}={\left(b_{i1},b_{i2},\cdots ,b_{im}\right)}^{T}$ is the normalised eigenvector of index *i* to sample *j*; $k_{c}=\left(k_{1c},k_{2c},\cdots ,k_{mc}\right)$ is the fuzzy class centre matrix of class *c*; *b*_ij_ is the normalised eigenvalue of the index *i* to sample *j*, 0≤*b*_ij_≤1; *k*_jc_ is the relative connection degree of the index *j* to the class *c*, 0≤*k*_jc_≤1. Because different factors have different weights, Eq. (7) is rewritten as Eq. (8).

(8) $$d=\left\|w_{j}\cdot \left(b_{i}-k_{c}\right)\right\|=\sqrt{\sum_{j=1}^{m}{\left[w_{j}\cdot \left(b_{ij}-k_{jc}\right)\right]}^{2}}$$

where *w*_j_ is the weight of the assessment factor *j*, which can be calibrated using the AHP approach; $w_{j}={\left(w_{1},w_{2},\cdots ,w_{m}\right)}^{T}$; and $\sum_{j=1}^{m}w_{j}=1$.

According to He et al. (2011) [11], the generalised weighted Euclidean distance (*D*) can be expressed by using Eq. (9).

(9) $$D=h_{ci}\cdot d=h_{ci}\cdot \left\|w_{j}\cdot \left(b_{i}-k_{c}\right)\right\|=h_{ci}\cdot \sqrt{\sum_{j=1}^{m}{\left[w_{j}\cdot \left(b_{ij}-k_{jc}\right)\right]}^{2}}$$

where *h*_ci_ is the relative connection degree of the sample *i* to the class *c*, 0≤*h*_ci_≤1, $\sum_{c=1}^{k}h_{ci}=1$. To make the assessment sample close to a given class standard, that is, to satisfy the minimum sum of squares of the generalised weight [12], the objective function can be constructed as Eq. (10).

(10) $$\min\left[F\left(w_{j},h_{ci},k_{jc}\right)\right]=\min\left\{\sum_{i=1}^{n}\sum_{c=1}^{k}{\left[h_{ci}\left\|w_{j}\cdot \left(b_{ij}-k_{jc}\right)\right\|\right]}^{2}\right\}$$

The boundary conditions are $\sum_{c=1}^{k}k_{ci}=1;\text{ 0}\leq k_{ci}\leq 1$. Similar to the derivation in literature (He et al., 2011) [3], the fuzzy clustering matrix (***H***) and fuzzy class centre matrix (***K***) can be obtained as shown in Eqs. (11), (12).

(11) $$H={\left(h_{ci}\right)}_{k\times n}={\left[\sum_{c=1}^{k}\frac{\sum_{j=1}^{m}{\left[w_{j}\left(b_{ij}-k_{jc}\right)\right]}^{2}}{\sum_{j=1}^{m}{\left[w_{j}\left(b_{ij}-k_{jc}\right)\right]}^{2}}\right]}^{-1}$$

(12) $$K={\left(k_{jc}\right)}_{m\times k}=\sum_{i=1}^{n}h_{ci}^{2}w_{j}^{2}b_{ij}/\sum_{i=1}^{n}h_{ci}^{2}w_{j}^{2}$$

After the adoption of the FCA method, the assessment sample can be classified into five classes, which can be found in the research article (Lyu et al., 2019) [1]. Based on the clustering matrix (***H***) and fuzzy class centre matrix (***K***), the combined FAHP-FCA can be applied to modify the weights from the AHP and the interval FAHP.
